# Dataset of liver proteins changed in eu- and hypothyroid female rats upon in vivo exposure to hexabromocyclododecane (HBCD)

**DOI:** 10.1016/j.dib.2016.02.047

**Published:** 2016-02-27

**Authors:** I. Miller, T. Serchi, S. Cambier, C. Diepenbroek, J. Renaut, J.H.J. van den Berg, C. Kwadijk, A.C. Gutleb, E. Rijntjes, A.J. Murk

**Affiliations:** a Institute for Medical Biochemistry, Department for Biomedical Sciences, University of Veterinary Medicine Vienna, Veterinaerplatz 1, A-1210 Vienna, Austria; b Environmental Research and Innovation (ERIN) Department, Luxembourg Institute of Science and Technology (LIST), 5, avenue des Hauts-Fourneaux, L-4362 Esch-sur-Alzette, Grand-duchy of Luxembourg; c Wageningen University, Human and Animal Physiology, P.O. Box 338, 6700 AH Wageningen, The Netherlands; d Charité-Universitätsmedizin Berlin, Institute for Experimental Endocrinology, Augustenburger Platz 1, 13353, Berlin, Germany; e Wageningen University, Division of Toxicology, Tuinlaan 5, 6703HE Wageningen, The Netherlands; f Wageningen Institute for Marine Resources & Ecosystem Studies, IMARES, IJmuiden, The Netherlands

**Keywords:** HBCD, Proteomics, Rat, Liver, Hypothyroidism, Lipid metabolism

## Abstract

Female Wistar rats with different thyroid status (eu-, hypothyroid) were exposed to 0, 3 or 30 mg/kg body weight of the flame retardant HBCD for 7 days. Changes in protein patterns obtained by 2D-DIGE were evaluated, and different animal groups compared taking into account their exposure and thyroid status. Proteins significantly altered in abundance in any of these comparisons were identified by mass spectrometry. These data, together with hormone data of the animals, are discussed in “Hexa-bromocyclododecane (HBCD) induced changes in the liver proteome of eu- and hypothyroid female rats” (Miller et al., 2016) [1].

**Specifications Table**

**Table:** 

Subject area	*Biology*
More specific subject area	*Environmental Toxicology*
Type of data	*Tables, image (annotated gel image)*
How data was acquired	2*D Fluorescence Difference Gel Electrophoresis (*2*D-DIGE) and mass spectrometry*
Data format	*Analyzed and filtered data*
Experimental factors	*Liver lysates of eu- and hypothyroid female rats differently exposed to HBCD*
Experimental features	*Comparative proteomic analysis of rat liver lysates using* 2*D-DIGE. Proteins present in differentially abundant protein spots (regarding HBCD exposure, amount, and thyroid status) were identified using MALDI TOF/TOF analysis.*
Data source location	*Origin of samples: Wageningen University, Wageningen, The Netherlands*
*Data collection: Luxembourg Institute of Science and Technology, Esch-sur-Alzette, Luxembourg*
Data accessibility	*MS- and regulation data is with this article as Supplementary material*

**Value of the data**

• Identification of liver proteins from female rats altered due to HBCD exposure.
• Identification of liver proteins from female rats changed in hypothyroid status.
• Data showing single and combined effects (HBCD exposure, hypothyroidism).
• Identified liver proteins form the basis for further studies to achieve a more detailed understanding of involved mechanism.

## 1 Data

Two-dimensional electrophoresis of liver protein lysates showed complex patterns of about 3000 spots per gel. Patterns of 24 gels from different exposures of eu- and hypothyroid rats were evaluated quantitatively. The data from different animals groups were compared, taking different aspects into account (HBCD exposure, thyroid status). Statistically significant fold-changes of at least 30% between groups (*P*<0.05 within group) were considered to be relevant.

**Fig. 1:**
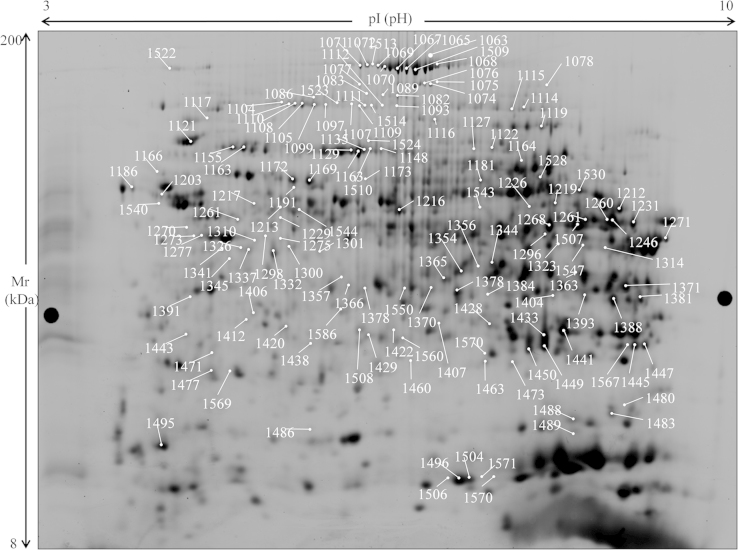
Image of a rat liver 2D-DIGE gel (master gel, grey level image). All spots with statistically significant abundance changes are labelled; spot numbers refer to identifications in Table 1. For details on protein identification see Supplemental Table 1, for data on protein abundance, see Supplemental Table 2.

The master gel is presented in Fig. 1, and all spots with significant abundance changes in any of the performed comparisons are labelled. Spot numbers refer to the protein identifications listed in Table 1 (peptide list in Supplemental Table 1), and to abundance changes in the various animal groups (Supplemental Table 2).

## 2 Experimental design, materials and methods

### 2.1 Animals, treatment and experimental protocol

The animal experiment was detailed in [1] and was approved under number 2007-041 by the Animal Welfare Committee of Wageningen University. In brief, female Wistar WU (HsdCpbWU) rats with normal or reduced thyroid function (hypothyroid) were orally exposed to 0, 3 or 30 mg/kg bw/d HBCD, respectively, for 7 consecutive days. Four liver samples per group were analyzed by proteomic methods.

### 2.2 Proteomic analysis

Two-dimensional fluorescence difference gel electrophoresis (2D-DIGE) was performed as previously described, with minor modifications [2], [3]. Rat livers were homogenized using the GE sample grinding kit in lysis buffer (urea 7 M; thiourea 2 M; CHAPS 2% w/w; tris 30 mM) containing protease inhibitor Complete Mini (Roche, Brussels, Belgium). Supernatants obtained after centrifugation (15 min at 30,000 g) were collected and stored at −20 °C until use. Protein concentration was determined according to Bradford [4]. Fifty µg per sample were labelled with CyDyes according to the manufacturer׳s instructions and separated on IPGs of a non-linear 3-10 pH-range. The second dimensional SDS-PAGE was performed in 12.5% precast gels (SERVA Electrophoresis GmbH, Heidelberg, Germany). Gel images (acquired on a Typhoon 9400) were analyzed with the DeCyder 7.0 software package (both GE Healthcare, Diegem, Belgium). Gels were matched and subjected to univariate and multivariate analysis in order to highlight differentially regulated spots (fold change at least 1.3) with a *P*-value in the respective univariate ANOVA or two way ANOVA <0.05.

Differentially abundant spots were automatically picked, tryptically digested and spotted on the MALDI target by the use of the Ettan *Spot* Handling Workstation (GE Healthcare, Diegem, Belgium). Protein identification was carried out on the Applied Biosystems MALDI-Tof-Tof 4800 Proteomics Analyser (Applied Biosystem, Gent, Belgium) as previously described [2]. Protein identification was performed by searching protein mass fingerprints (PMF) and MS/MS spectra against the SwissProt database with “*Rattus norvegicus*” as taxonomy. Searches were performed using the ProteinPilot software (Sciex, Nieuwerkerk aan den Ijssel, The Netherlands) and the searching algorithm MASCOT (Matrix Science, www.matrixscience.com, London, UK). For each spot one protein mass fingerprint and up to 8 MS/MS spectra were generated. Parameters for the search were set as follow: up to two missed cleavages allowed, 100 ppm tolerance in PMF, 0.75 Da mass tolerance for precursor ion mass, carbamidomethyl cysteine as fixed modification, oxidation of methionine and oxidation of tryptophan (single oxidation, double oxidation and kynurenin) as variable modifications. Identifications were considered to be significant when the combined MOWSE score had *P*<0.05.

Statistics, including univariate analysis (ANOVA and *t*-test) and multivariate analysis (two way ANOVA), was performed using the Extended Data Analysis (EDA) module, which is present inside the Decyder 7.0 software package.

## Figures and Tables

**Table 1:** Proteins of the present dataset, identified by MALDI-TOF/TOF analysis.

**Spot number**	**Protein name**	**Species**	**Swiss-Prot Acc. N°**
**1063, 1065, 1067–1071, 1074–1076, 1078**	Carbamoyl-phosphate synthase[ammonia], mitochondrial	Rattus norvegicus	CPSM_RAT
**1072**	Murinoglobulin-2	Rattus norvegicus	MUG2_RAT
**1077, 1082, 1083**	Pyruvate carboxylase, mitochondrial	Rattus norvegicus	PYC_RAT
**1086**	ATP-citrate synthase	Rattus norvegicus	ACLY_RAT
**1089**	C-1-tetrahydrofolate synthase, cytoplasmic	Rattus norvegicus	C1TC_RAT
**1093**	Alpha-aminoadipic semialdehyde synthase, mitochondrial	Rattus norvegicus	AASS_RAT
**1094, 1100**	2-oxoglutarate dehydrogenase, mitochondrial	Rattus norvegicus	ODO1_RAT
**1099, 1105, 1107–1110, 1114**	Aldehyde dehydrogenase family1 member L1	Rattus norvegicus	AL1L1_RAT
**1111**	Aldehyde dehydrogenase1 family, member L2	Mus musculus	gi|21961590
**1112, 1115–1117, 1119**	Sarcosine dehydrogenase, mitochondrial	Rattus norvegicus	SARDH_RAT
**1121, 1122**	Elongation factor2	Rattus norvegicus	EF2_RAT
**1123**	Cytoplasmic aconitate hydratase	Rattus norvegicus	ACOC_RAT
**1129**	Dimethylglycine dehydrogenase, mitochondrial	Rattus norvegicus	M2GD_RAT
**1135**	Serotransferrin	Rattus norvegicus	TRFE_RAT
**1148**	Propionyl-CoA carboxylase alpha chain, mitochondrial	Rattus norvegicus	PCCA_RAT
**1155**	78kDa glucose-regulated protein	Rattus norvegicus	GRP78_RAT
**1161, 1165**	Heat shock cognate 71 kDa protein	Rattus norvegicus	HSP7C_RAT
**1163, 1164**	rCG56002	Rattus norvegicus	gi|149036727
**1169, 1172, 1173, 1181, 1186**	Serum albumin	Rattus norvegicus	ALBU_RAT
**1191**	Delta-1-pyrroline-5-carboxylate dehydrogenase, mitochondrial	Cricetulus griseus	gi|344249754
**1203**	UV excision repair protein RAD23 homolog B	Rattus norvegicus	RD23B_RAT
**1212**	PREDICTED: aldehyde dehydrogenase 8 family, member A1-like isoform 2	Rattus norvegicus	gi|109460389
**1213**	Pyruvatekinase isozymes R/L	Rattus norvegicus	KPYR_RAT
**1216, 1219**	Proteindisulfide-isomerase A3	Rattus norvegicus	PDIA3_RAT
**1217**	Liver carboxylesterase 4	Rattus norvegicus	EST4_RAT
**1226**	Formimidoyl transferase-cyclodeaminase	Rattus norvegicus	FTCD_RAT
**1229**	Calreticulin	Rattus norvegicus	CALR_RAT
**1231**	Methylmalonate-semialdehyde dehydrogenase[acylating], mitochondrial	Rattus norvegicus	MMSA_RAT
**1246**	Alpha-1-antiproteinase	Rattus norvegicus	A1AT_RAT
**1260, 1268**	Alanine-glyoxylate aminotransferase 2, mitochondrial	Rattus norvegicus	AGT2_RAT
**1261**	Glutathione synthetase	Rattus norvegicus	GSHB_RAT
**1262**	4-trimethylaminobutyraldehyde dehydrogenase	Rattus norvegicus	AL9A1_RAT
**1270, 1277**	Phenylalanine-4-hydroxylase	Rattus norvegicus	PH4H_RAT
**1271**	Succinate-semialdehyde dehydrogenase, mitochondrial	Rattus norvegicus	SSDH_RAT
**1273**	Hydroxymethylglutaryl-CoA synthase, mitochondrial	Rattus norvegicus	HMCS2_RAT
**1275**	Alpha-enolase	Rattus norvegicus	ENOA_RAT
**1296**	Ifi47 protein	Rattus norvegicus	gi|44890246
**1298, 1301, 1310**	Betaine--homocysteine S-methyltransferase 1	Rattus norvegicus	BHMT1_RAT
**1300**	Eukaryotic initiation factor 4A-II	Rattus norvegicus	IF4A2_RAT
**1314**	3-ketoacyl-CoA thiolase, mitochondrial	Rattus norvegicus	THIM_RAT
**1323, 1326**	Argininosuccinate synthase	Rattus norvegicus	ASSY_RAT
**1332**	Keratin, type I cytoskeletal 18	Rattus norvegicus	K1C18_RAT
**1337**	Aspartate aminotransferase, cytoplasmic	Rattus norvegicus	AATC_RAT
**1341, 1345, 1354**	Actin, cytoplasmic 1	Rattus norvegicus	ACTB_RAT
**1344**	Creatinekinase B-type	Rattus norvegicus	KCRB_RAT
**1356**	Aspartate aminotransferase, mitochondrial	Rattus norvegicus	AATM_RAT
**1357**	Serum paraoxonase/arylesterase2	Rattus norvegicus	PON2_RAT
**1363, 1365**	Fructose-bisphosphate aldolase B	Rattus norvegicus	ALDOB_RAT
**1366**	Serum paraoxonase/lactonase 3	Rattus norvegicus	PON3_RAT
**1370, 1371, 1374, 1378, 1384**	Fructose-1,6-bisphosphatase 1	Rattus norvegicus	F16P1_RAT
**1381**	Adipocyte plasmamembrane-associated protein	Rattus norvegicus	APMAP_RAT
**1388**	Farnesyl pyrophosphate synthase	Rattus norvegicus	FPPS_RAT
**1391, 1393**	Arginase-1	Rattus norvegicus	ARGI1_RAT
**1404, 1417**	3-oxo-5-beta-steroid 4-dehydrogenase	Rattus norvegicus	AK1D1_RAT
**1406**	Glyceraldehyde-3-phosphate dehydrogenase	Rattus norvegicus	G3P_RAT
**1412**	3-alpha-hydroxy steroid dehydrogenase	Rattus norvegicus	DIDH_RAT
**1420, 1429**	Glycerol-3-phosphate dehydrogenase[NAD+], cytoplasmic	Rattus norvegicus	GPDA_RAT
**1422**	L-lactate dehydrogenase A chain	Rattus norvegicus	LDHA_RAT
**1428**	Beta-lactamase-like protein 2	Rattus norvegicus	LACB2_RAT
**1433**	Ester hydrolase C11 orf 54 homolog	Rattus norvegicus	CK054_RAT
**1438**	Sulfotransferase 1A1	Rattus norvegicus	ST1A1_RAT
**1441, 1443**	Thiosulfate sulfurtransferase	Rattus norvegicus	THTR_RAT
**1445**	Guanine nucleotide-binding protein subunit beta-2-like1	Rattus norvegicus	GBLP_RAT
**1447**	Regucalcin	Rattus norvegicus	RGN_RAT
**1449**	D-beta-hydroxybutyrate dehydrogenase, mitochondrial	Rattus norvegicus	BDH_RAT
**1450**	Hydroxyacyl-coenzyme A dehydrogenase, mitochondrial	Rattus norvegicus	HCDH_RAT
**1460**	Nitrilase homolog 1	Rattus norvegicus	NIT1_RAT
**1463**	Proteasome activator complex subunit1	Rattus norvegicus	PSME1_RAT
**1471**	Nicotinate-nucleotide pyrophosphorylase [carboxylating]	Rattus norvegicus	NADC_RAT
**1473**	Thiopurine S-methyltransferase	Rattus norvegicus	TPMT_RAT
**1477, 1483**	Electron transfer flavoprotein subunit beta	Rattus norvegicus	ETFB_RAT
**1480**	Isoamyl acetate-hydrolyzing esterase 1 homolog	Rattus norvegicus	IAH1_RAT
**1486**	Glutathione S-transferase Mu2	Rattus norvegicus	GSTM2_RAT
**1488**	Glutathione S-transferase alpha-5	Rattus norvegicus	GSTA5_RAT
**1489**	Peroxiredoxin-4	Rattus norvegicus	PRDX4_RAT
**1495**	protein ETHE1, mitochondrial	Rattus norvegicus	gi|157819563
**1496, 1509, 1510**	Carbonic anhydrase 3	Rattus norvegicus	CAH3_RAT
**1504**	Endoplasmic reticulum resident protein 29	Rattus norvegicus	ERP29_RAT
**1506**	Glutathione S-transferase alpha-1	Rattus norvegicus	GSTA1_RAT
**1507**	Glutathione S-transferase alpha-2	Rattus norvegicus	GSTA2_RAT
**1508**	Glutathione S-transferase alpha-3	Rattus norvegicus	GSTA3_RAT
**1512**	Glutathione S-transferase alpha-4	Rattus norvegicus	GSTA4_RAT
**1514**	NADH dehydrogenase [ubiquinone] flavoprotein 2, mitochondrial	Rattus norvegicus	NDUV2_RAT
**1522**	Glutathione S-transferase P	Rattus norvegicus	GSTP1_RAT
**1523**	biliverdin reductase B (flavinreductase(NADPH)) (predicted), isoform CRA_c	Rattus norvegicus	gi|149056527
**1524**	Peroxiredoxin-1	Rattus norvegicus	PRDX1_RAT
**1528, 1530**	Abhydrolase domain-containing protein 14B	Rattus norvegicus	ABHEB_RAT
**1540**	Peptidyl-prolyl cis-trans isomerase F, mitochondrial	Rattus norvegicus	PPIF_RAT
**1543**	Cofilin-1	Rattus norvegicus	COF1_RAT
**1544**	Peptidyl-prolyl cis-trans isomerase A	Rattus norvegicus	PPIA_RAT
**1547**	Low molecular weight phosphotyrine protein phosphatase	Rattus norvegicus	PPAC_RAT
**1550**	Ubiquitin-conjugating enzyme E2D2	Rattus norvegicus	UB2D2_RAT
**1560**	Cytochrome b5	Rattus norvegicus	CYB5_RAT
**1567–1569**	Hemoglobin subunit alpha-1/2	Rattus norvegicus	HBA_RAT
**1570, 1571**	Fatty acid-binding protein, liver	Rattus norvegicus	FABPL_RAT
**1586**	Enoyl-CoA hydratase, mitochondrial	Rattus norvegicus	ECHM_RAT

## References

[1] Miller I., Serchi T., Cambier S., Diepenbroek C., Renaut J., Van den Berg J.H.J., Kwadijk C., Gutleb A.C., Rijntjes E., Murk A.J. (2016). Hexa bromocyclododecane (HBCD) induced changes in the liver proteome of eu- and hypothyroid female rats. Toxicol. Lett..

[2] Haas B., Serchi T., Wagner D.R., Gilson G., Planchon S., Renaut J., Hoffmann L., Bohn T., Devaux Y. (2011). Proteomic analysis of plasma samples from patients with acute myocardial infarction identifies haptoglobin as a potential prognostic biomarker. J. Proteom..

[3] Pasquali M., Serchi T., Renaut J., Hoffmann L., Bohn T. (2013). 2D difference gel electrophoresis reference map of a Fusarium graminearum nivalenol producing strain. Electrophoresis.

[4] Bradford M.M. (1976). A rapid and sensitive method for the quantitation of microgram quantities of protein utilizing the principle of protein-dye binding. Anal. Biochem..

