# Supplementary material for: Dataset of liver proteins changed in eu- and hypothyroid female rats upon in vivo exposure to hexabromocyclododecane (HBCD)
Source: Data Brief. 2016 Feb 27;7:386–92. doi: 10.1016/j.dib.2016.02.047 (PMC4781926; doi:10.1016/j.dib.2016.02.047)
Supplement: Supplementary file 1 — Supplementary material [file mmc1.doc]

None of the authors has a conflict of interest. Conflict of interest forms for all single authors have already been included in the accompanying manuscript in Toxicology Letter.

Ingrid Miller (on behalf of all authors)
